# Con7 is a key transcription regulator for conidiogenesis in the plant pathogenic fungus *Fusarium graminearum*

**DOI:** 10.1128/msphere.00818-23

**Published:** 2024-04-09

**Authors:** Soobin Shin, Jiyeun Park, Lin Yang, Hun Kim, Gyung Ja Choi, Yin-Won Lee, Jung-Eun Kim, Hokyoung Son

**Affiliations:** 1 Department of Agricultural Biotechnology, Seoul National University, Seoul, South Korea; 2 The Provincial Key Lab of Plant Pathology of Hubei Province, College of Plant Science and Technology, Huazhong Agricultural University, Wuhan, Hubei, China; 3 Eco-friendly New Materials Research Group, Research Center for Biobased Chemistry, Division of Convergence Chemistry, Korea Research Institute of Chemical Technology, Daejeon, South Korea; 4 Research Institute of Climate Change and Agriculture, National Institute of Horticultural and Herbal Science, Jeju, South Korea; 5 Research Institute of Agriculture and Life Sciences, Seoul National University, Seoul, South Korea; CNRS-INSERM-Université Côte d'Azur, Nice, France

**Keywords:** *CON7*, *Fusarium graminearum*, conidiation, chitin synthesis

## Abstract

The mycelium of the plant pathogenic fungus *Fusarium graminearum* exhibits distinct structures for vegetative growth, asexual sporulation, sexual development, virulence, and chlamydospore formation. These structures are vital for the survival and pathogenicity of the fungus, necessitating precise regulation based on environmental cues. Initially identified in *Magnaporthe oryzae*, the transcription factor Con7p regulates conidiation and infection-related morphogenesis, but not vegetative growth. We characterized the Con7p ortholog *Fg*Con7, and deletion of *FgCON7* resulted in severe defects in conidium production, virulence, sexual development, and vegetative growth. The mycelia of the deletion mutant transformed into chlamydospore-like structures with high chitin level accumulation. Notably, boosting *FgABAA* expression partially alleviated developmental issues in the *FgCON7* deletion mutant. Chromatin immunoprecipitation (ChIP)–quantitative PCR (qPCR) analysis confirmed a direct genetic link between *FgABAA* and *FgCON7*. Furthermore, the chitin synthase gene *Fg6550* (FGSG_06550) showed significant upregulation in the *FgCON7* deletion mutant, and altering *FgCON7* expression affected cell wall integrity. Further research will focus on understanding the behavior of the chitin synthase gene and its regulation by *Fg*Con7 in *F. graminearum*. This study contributes significantly to our understanding of the genetic pathways that regulate hyphal differentiation and conidiation in this plant pathogenic fungus.

**IMPORTANCE:**

The ascomycete fungus *Fusarium graminearum* is the primary cause of head blight disease in wheat and barley, as well as ear and stalk rot in maize. Given the importance of conidia and ascospores in the disease cycle of *F. graminearum*, precise spatiotemporal regulation of these biological processes is crucial. In this study, we characterized the *Magnaporthe oryzae* Con7p ortholog and discovered that *Fg*Con7 significantly influences various crucial aspects of fungal development and pathogenicity. Notably, overexpression of *FgABAA* partially restored developmental defects in the *FgCON7* deletion mutant. ChIP-qPCR analysis confirmed a direct genetic link between *FgABAA* and *FgCON7*. Furthermore, our research revealed a clear correlation between *Fg*Con7 and chitin accumulation and the expression of chitin synthase genes. These findings offer valuable insights into the genetic mechanisms regulating conidiation and the significance of mycelial differentiation in this plant pathogenic fungus.

## INTRODUCTION

In plant pathogenic fungi, successful pathogenesis involves a sequence of infection stages, including attachment of spores to host surfaces, spore germination, and penetration into plant tissue (1). Throughout the plant infection and disease cycle, fungal cells undergo specific differentiation to specific structures for vegetative growth, asexual sporulation, sexual reproduction, virulence, and chlamydospore formation. Multiple genes participate in these biological processes, which are controlled by transcription factors (TFs). TFs play a crucial role in regulating the expression of target genes that drive cellular and developmental responses to environmental signals (2). Investigating TF genes through either gene disruption or overexpression provides valuable insights into understanding their functions and the interconnected relationships among TFs, which contribute to cellular differentiation and development (2).

The ascomycete fungus *Fusarium graminearum* is the primary cause of head blight disease in wheat and barley, along with ear and stalk rot in maize (3). Severe outbreaks of these diseases result not only in reduced yields but also in mycotoxin contamination on affected grains, posing acute and chronic health risks to humans and animals (4, 5). During its life cycle, *F. graminearum* generates both sexual (ascospores) and asexual (conidia) spores, serving as the principal inoculum sources of disease. Ascospores develop in ephemeral perithecia on infected plant residues and disperse into the air (6–8). Conidia, originating from sporodochia on diseased crops, facilitates secondary infection (7). Additionally, asexual resting cells, including chlamydospores and chlamydospore-like structures, have been proposed as potential alternative survival structures (3, 9, 10). Considering the significance of conidia and ascospores in the disease cycle of *F. graminearum*, the precise spatiotemporal regulation of these biological processes is essential.

The TF *CON7* gene was initially discovered in the rice blast fungus *Magnaporthe oryzae* as a pivotal regulator affecting infection-related morphogenesis. Deletion of the *CON7* gene in *M. oryzae* resulted in defective conidium morphology and the failure of appressorium formation without affecting mycelial growth (11–13). Disruption of the gene encoding the Con7 homolog in *F. oxysporum* resulted in several defects in hyphal growth, conidiation, and cell wall morphology (14). In *F. graminearum*, the homeobox TF *Fg*Htf1 promotes conidiation and activates the expression of *FgCON7* and of other conidiation-related genes (15). Overall, Con7 is anticipated to have a significant role in conidium morphology and production, although its specific function might vary among different species.

While previous studies have found the association of Con7 withn conidial morphology and production, the exploration of Con7’s regulatory mechanisms remains limited. Hence, based on our large-scale functional study, we hypothesized that the Con7 ortholog might serve roles beyond conidiation in *F. graminearum* (2). In this study, we focused on the Con7 ortholog, termed *Fg*Con7, in *F. graminearum*. Through genetic manipulations, including deletion and overexpression of *FgCON7*, we showed that *Fg*Con7 not only affects conidiation but also modulates various developmental processes, including mycelia growth, sexual development, chlamydospore-like structure formation, and chitin synthesis. Furthermore, our findings revealed that *Fg*Con7 is essential for conidiation as it directly regulates master regulator genes of conidiation. The results of this study contribute to our understanding of mycelial differentiation in plant pathogenic fungi.

## RESULTS

### Identification of C_2_H_2_ zinc finger transcription factor *Fg*Con7 in *F. graminearum*

**Fig 1:**
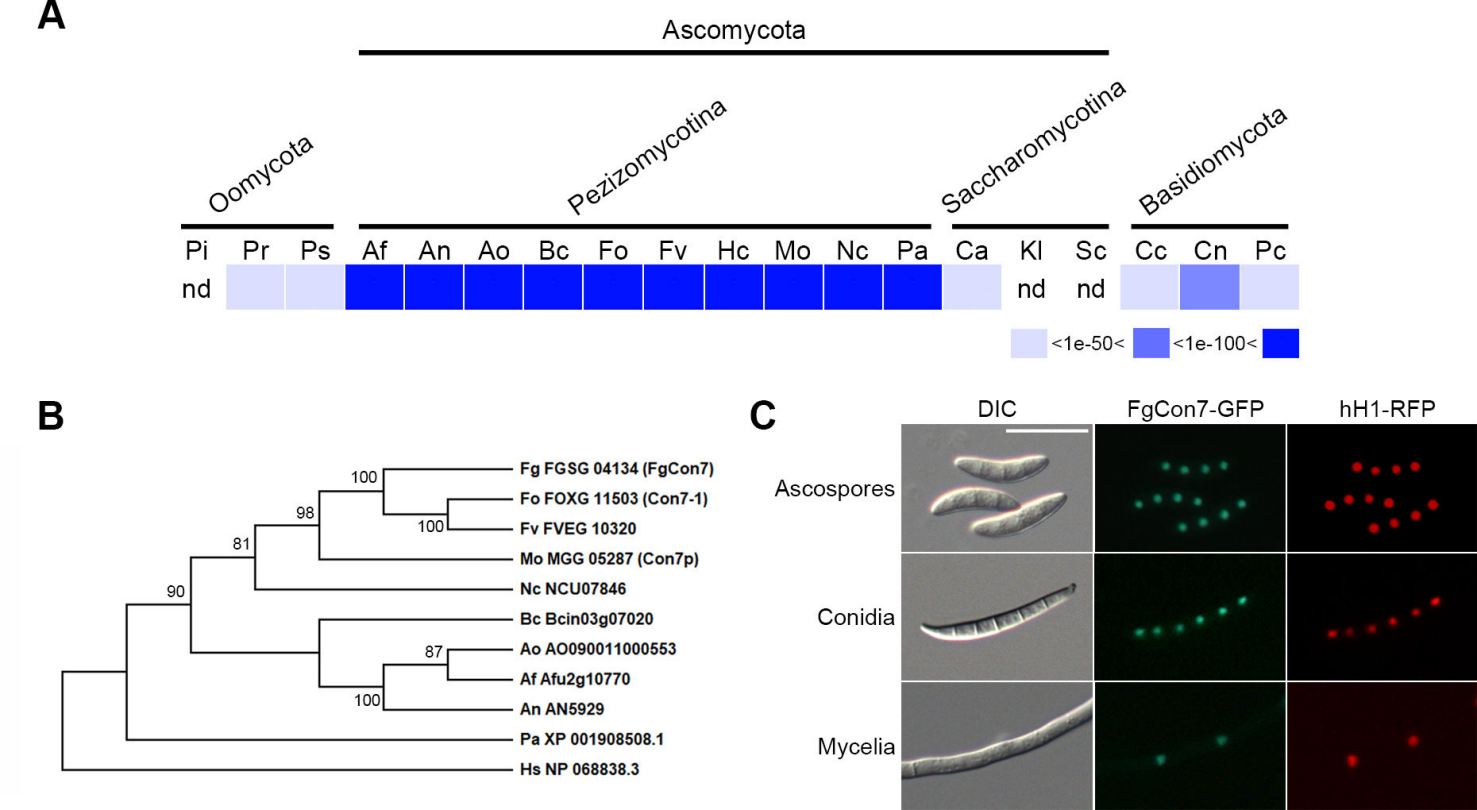
Distribution and phylogenetic analysis of Con7 homologs in fungi. (**A**) Distribution of Con7 homologs in representative fungal species. The distribution image was constructed by using the BLASTMatrix tool that is available on the Comparative Fungal Genomics Platform (http://cfgp.riceblast.snu.ac.kr/) (17). (**B**) Phylogenetic tree of Con7 homologs. Amino acid sequences were aligned using ClustalW, and the MEGA X software was used to perform phylogenetic analysis using the neighbor-joining method with 1,000 bootstrap replicates. Pi, *Phytophthora infestans*; Pr, *P. ramorum*; Ps, *P. sojae*; Af, *Aspergillus fumigatus*; An, *A. nidulans*; Ao, *A. oryzae*; Bs, *Botrytis cinerea*; Fo, *Fusarium oxysporum*; Fv, *F. verticillioides*; Hs, *Histoplasma capsulatum*; Mo, *Magnaporthe oryzae*; Nc, *Neurospora crassa*; Pa, *Podospora anserine*; Ca, *Candida albicans*; Kl, *Kluyveromyces lactis*; Sc, *Saccharomyces cerevisiae*; Cc, *Coprinus cinereus*; Cn, *Cryptococcus neoformans*; Pc, *Phanerochaete chrysosporium*; nd, not detected. (**C**) Cellular localization of *Fg*Con7. *Fg*Con7 was fused with green fluorescent protein (GFP), and histone H1 was fused with red fluorescent protein (RFP). Scale bar = 20 µm.

In our prior genome-wide functional analyses of TFs in *F. graminearum* (2), we identified FGRAMPH1_01G14573 (previously FGSG_04134) as the *M. oryzae CON7* ortholog (*MoCON7*), with *Fg*Con7 protein sharing 55% identity with *Mo*Con7 (13). *Fg*Con7 consists of a C2H2-type zinc finger DNA-binding domain (InterPro: IPR007087, zinc finger, C2H2-type) and possesses a distinct nuclear localization signal (NLS) (304-IRKEWKQRKKEEEA-317), such as *Mo*Con7 (Fig. S1) (16). *Fg*Con7 is highly conserved among fungal species within the subphylum Pezizomycotina of Ascomycota (Fig. 1A) and exhibits a close phylogenetic relationship with *F. oxysporum* (identity: 88%) and *F. verticillioides* orthologs (identity: 89%) (Fig. 1B).

To assess the function of *Fg*Con7 on both physiological and pathological aspects of *F. graminearum*, we generated deletion mutants by replacing *FgCON7* with a geneticin resistance gene cassette (*GEN*) in the wild-type strain. Furthermore, we fused the *FgCON7* open-reading frame (ORF) with the green fluorescent protein-encoding gene (*GFP*) under its native promoter and introduced it into the Δ*Fgcon7* strain, resulting in the production of a complementation strain (FgCON7c). To validate previous results and further investigate the functions of *FgCON7*, we generated the FgCON7c-r strain (Δ*Fgcon7::FgCON7-GFP-HYG hH1::hH1-RFP-GEN*) through an outcross between the mat1r (18) and FgCON7c strains. This was done to ascertain the nuclear localization of *Fg*Con7. *Fg*Con7-GFP showed distinct co-localization with hH1-RFP throughout observed developmental stages, including ascospores, conidia, and mycelia (Fig. 1C). Findings from both phylogenetic and co-localization analyses showed that *Fg*Con7 shares close relations with Con7 proteins found in Pezizomycotina, harboring plant pathogenic fungi such as *M. oryzae* and *Fusarium* species.

### *FgCON7* contributes to vegetative growth, sexual reproduction, and virulence

Plant pathogenic fungi such as *M. oryzae* and *F. oxysporum* exhibit numerous developmental defects upon disruption of the *CON7* genes (14). To investigate whether similar effects occur in *F. graminearum*, we created various mutants, including the *FgCON7* deletion mutant (Δ*Fgcon7*) and complementation (FgCON7c) strains. Additionally, we conducted interspecies complementation by introducing the *CON7* ORF of *M. oryzae*, generating seven complemented mutants without ectopic integration (MoCON7c) (Fig. S2B). Furthermore, we generated *FgCON7* overexpression transformants (FgCON7oe), where *FgCON7* is regulated by a strong promoter (*P_EF1α_*) (Fig. S2C). The FgCON7oe mutants exhibited a transcript accumulation that was about 20-fold higher than that in the wild-type strain (Fig. S2D). We confirmed all genetic manipulations through Southern blot hybridizations (Fig. S2B through D).

**Fig 2:**
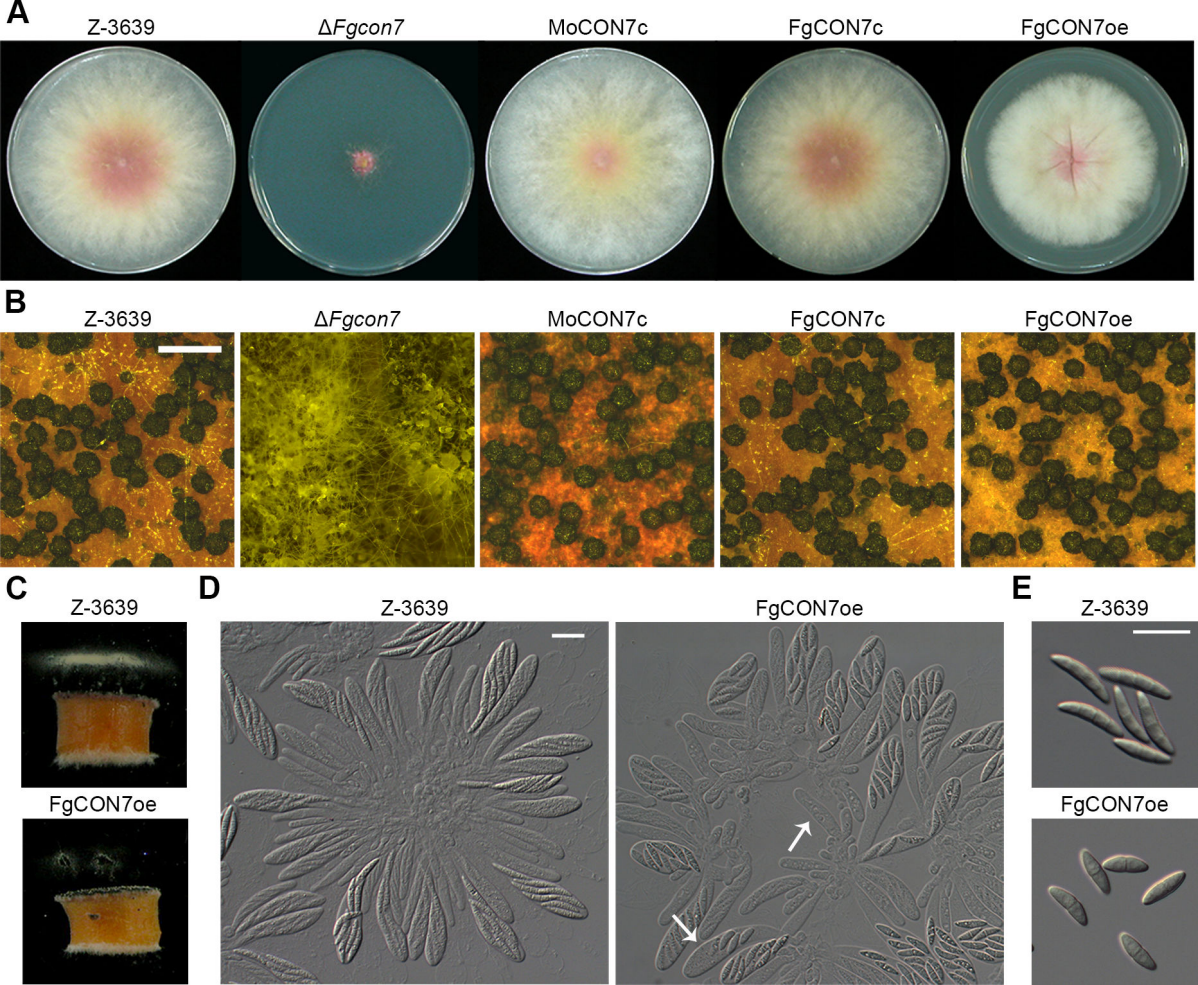
Mycelial growth and sexual development of *F. graminearum* strains. (**A**) Mycelial growth of *F. graminearum* strains on the complete medium (CM). Pictures were taken 5 days after inoculation. (**B**) Sexual development of *F. graminearum* strains on carrot agar medium. Dissection microscopic pictures were taken 10 days after sexual induction. Scale bar = 0.5 mm (**C**) Forcible ascospore discharge of the wild-type and *FgCON7* overexpression mutant strains (FgCON7oe). Photographs were taken 2 days after the assay was initiated. A semicircular agar block covered with perithecia was placed on a coverslip in the chamber, and the ascospores were allowed to be discharged horizontally. (**D**) Asci rosettes from the wild-type and FgCON7oe strains. The microscopic picture was taken 10 days after sexual induction. Scale bar = 20 µm. (**E**) Ascospore morphology. The microscopic picture was taken 10 days after sexual induction. Scale bar = 20 µm. WT, *F. graminearum* wild-type strain Z-3639; ∆*Fgcon7*, *FgCON7* deletion mutant; MgCON7c, ∆*Fgcon7*-derived strain complemented with CON7 of *Magnaporthe oryzae*; FgCON7c, ∆*Fgcon7*-derived strain complemented with *FgCON7-GFP*; FgCON7oe, transgenic strain that has the *EF1 α* promoter in place of the *FgCON7* promoter region.

**Fig 3:**
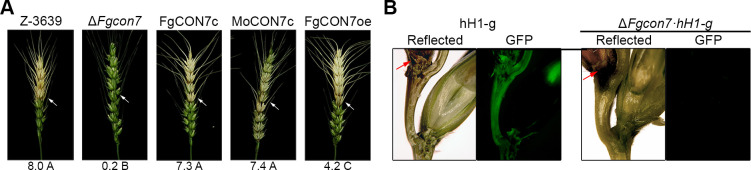
Virulence of *F. graminearum* strains. (**A**) Virulence on wheat heads. A center spikelet of each wheat head was injected with 10 µL of the conidia suspension. Mock, negative control mock inoculated with 0.01% of Tween 20. The disease index (diseased spikelets per wheat head) is denoted below the picture, and the values with different letters are significantly different (*P* < 0.05) based on Tukey’s HSD test. Mock, mock inoculation was performed with 0.01% Tween 20. Each arrow indicates the inoculation site for *F. graminearum* strains. (**B**) Micrographs of manually generated sections after infection of wheat heads. Wheat spikelets were inoculated with conidial suspensions of cytosolic GFP-expressing strains (hH1-g and *ΔFgcon7 · hH1-g*). Infected wheat heads were longitudinally dissected at 6 days after inoculation and observed by fluorescence microscopy. The GFP fluorescence signal indicates spreading of hyphae from the inoculation points. Arrowheads represent the inoculated spikelets. Reflected, bright field image. Each arrow indicates the inoculation site for *F. graminearum* strains.

While the deletion of *MoCON7* did not impair mycelial growth (13), deleting the *CON7* gene in *F. graminearum* led to significant deficiencies in vegetative growth on the complete medium (CM) (Fig. 2A). Interestingly, overexpressing *FgCON7* altered the morphology of mycelial colonies and slightly reduced vegetative growth compared to the wild-type strain (Fig. 2A). Concerning sexual development, Δ*Fgcon7* mutants were unable to produce any perithecium and exhibited increased pigment production on carrot agar (Fig. 2B). FgCON7oe mutants generated normally melanized perithecia, but they had defects in ascospore discharge due to abnormal production of asci and ascospores (Fig. 2B through E). FgCON7oe mutants displayed irregular rosette asci, with some containing fewer than eight ascospores (Fig. 2D). Furthermore, FgCON7oe mutants produced two-celled ascospores, unlike the wild-type strain, which produced four-celled ascospores (Fig. 2E). The virulence of each strain was assessed via point inoculation on flowering wheat heads. Notably, Δ*Fgcon7* mutants did not induce any blight symptoms, even in the inoculated spikelets, while the wild-type, FgCON7c, MoCON7c, and FgCON7oe strains exhibited typical head blight symptoms 21 days post-inoculation (Fig. 3A). Furthermore, we generated a Δ*Fgcon7·hH1-g* strain (Δ*Fgcon7::GEN hH1::hH1-GFP-GEN*) through a cross between the hH1-g (19) and Δ*Fgcon7* strains to observe mycelial movement on wheat heads during infection. Six days after inoculation, hyphae from hH1-g strains, possessing the wild-type allele of *FgCON7* and expressing cytosolic GFP, had spread to adjacent spikelets through rachis nodes from the inoculated spikelet. In contrast, Δ*Fgcon7·hH1-g* strains did not survive in the inoculation points and failed to spread to neighboring spikelets (Fig. 3B).

The complementation strain FgCON7c effectively restored the majority of the phenotypes observed in the deletion mutant. Furthermore, *MoCON7* also rescued the mutant phenotypes observed in Δ*Fgcon7* strains of *F. graminearum*. Collectively, these results highlight the essential role of Con7 in *F. graminearum* for vegetative growth, sexual development, and virulence. This suggests that Con7 in plant pathogenic fungi functions as a pivotal TF involved in various fungal developmental processes.

### *FgCON7* is essential for conidiogenesis and chlamydospore-like structure formation

**Fig 4:**
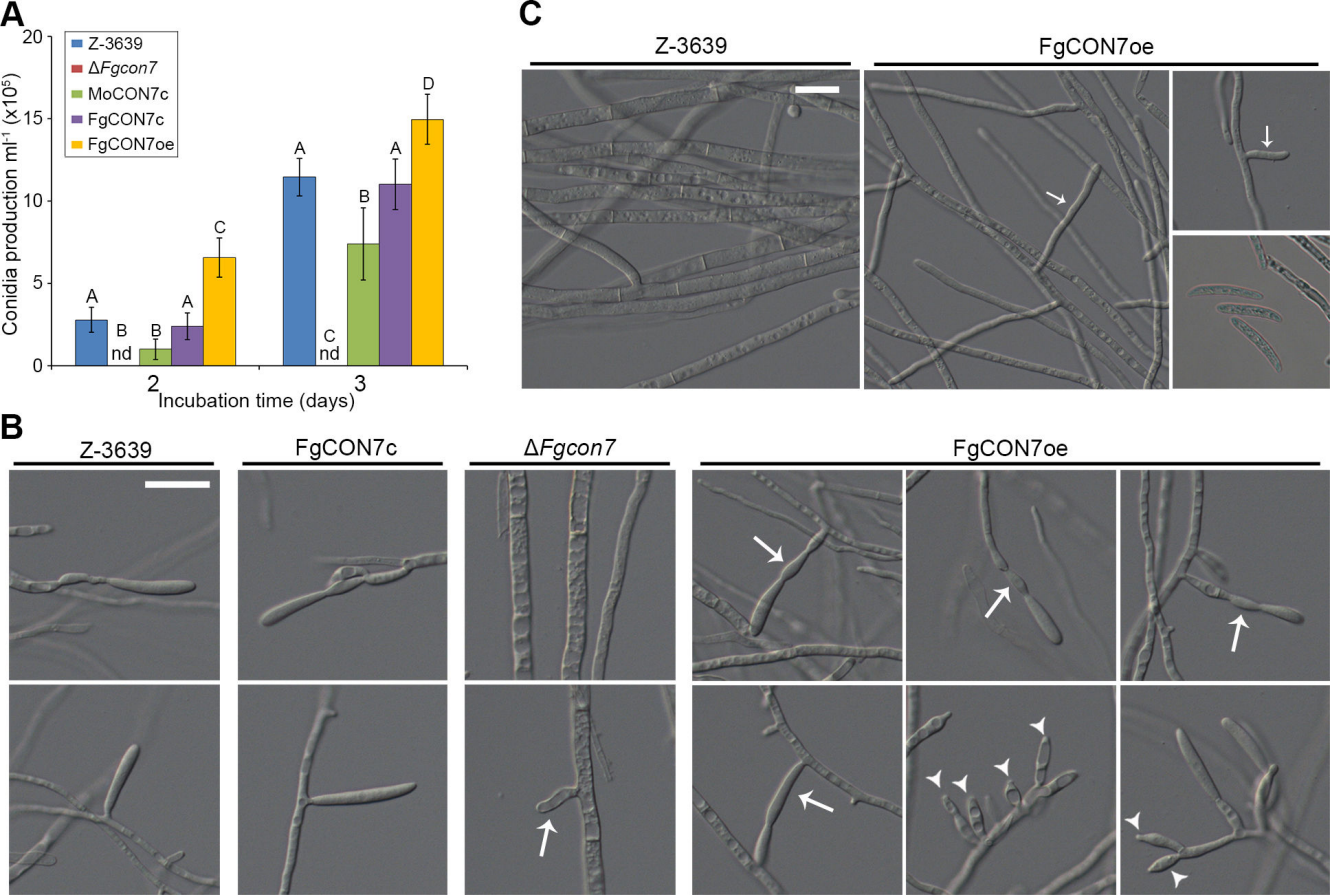
Asexual development in *F. graminearum* strains. (**A**) Conidiophore formation. Microscopic observation was performed 3 days after inoculation in CMC. Arrows and arrow heads indicate extended and overproduced conidiophores, respectively. Scale bar = 20 µm. (**B**) Quantification of conidium production in *F. graminearum* strains. Conidium production was induced in CMC media, and the number of each strain was measured 2 and 3 days after inoculation. Values with different letters are significantly different (*P* < 0.05) based on Tukey’s HSD test. nd: not detected. (**C**) Hyphal morphology of *F. graminearum* strains in CM. Mycelia of the *FgCON7* overexpression mutant were more frequently branched (arrows), and some conidia were produced (arrow heads). Scale bar = 20 µm.

As conidia play a crucial role in secondary infection during disease development, we observed asexual development. Δ*Fgcon7* strains exhibited immature conidiophores within their hyphae (black arrow in Fig. 4A). They failed to produce any conidia in the carboxymethyl cellulose medium (Fig. 4A). Conversely, *FgCON7* overexpression triggered the formation of excessive conidiophores (white arrowheads in Fig. 4A) and enhanced conidium production (Fig. 4B) compared to the wild-type strain. Some phialide cells in FgCON7oe appeared longer than those of the wild-type strain and abnormally shaped (white arrows in Fig. 4B). Moreover, *FgCON7* overexpression mutants displayed robust conidiophores and conidium production in CM, while the wild-type and complementation strains showed suppressed conidium production (Fig. 4C). However, many of the produced conidiophores seemed to develop as hyphae, suggesting that the overexpression of *FgCON7* amplified the initial stage of conidiogenesis.

**Fig 5:**
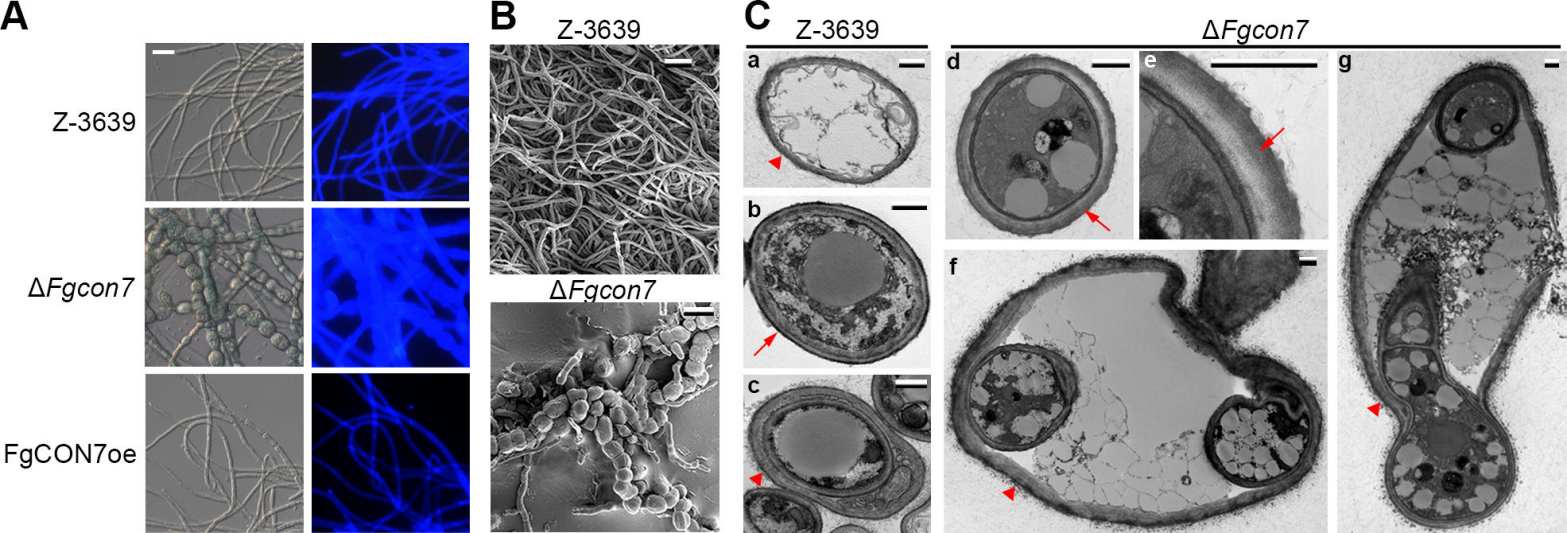
*FgCON7* is involved in chlamydospore formation. (**A**) Histological visualization of mycelia. Chitins were stained with calcofluor white. (**B**) Scanning electron microscopy (SEM) images of mycelia. Each strain was cultured in minimal conversion media supplemented with mannitol for 7 days. (**C**) Transmission electron microscopy (TEM) images of mycelia. Each strain was cultured in minimal conversion media supplemented with mannitol for 7 days. Scale bar = 0.5 µm. Each arrow indicates either single (arrow head)- or double (arrow)-layered cell walls in *F. graminearum* strains.

We then investigated whether *FgCON7* plays a role in formation of chlamydospore and chlamydospore-like structure. In cultures grown in minimal conversion media supplemented with mannitol (MMCM) to induce chlamydospore-like structures (20), Δ*Fgcon7* strains exhibited balloon-shaped hyphae with thick cell walls (Fig. 5A and B). Detailed histological analyses asserted that hyphal chlamydospores accumulated much more chitin than the normal hyphae of the wild-type strain (Fig. 5A). To confirm the presence of a double-layered cell wall, we conducted transmission electron microscopy observations. As previously described (21), older mycelia of the wild-type strain exhibited intrahyphal hyphae (Fig. 5C a–c). However, younger mycelia of the Δ*Fgcon7* strains showed considerably thicker cell walls than those of the wild-type strain, where some cell walls were distinctly double-layered (Fig. 5C d–e). The deletion of *FgCON7* not only resulted in thicker cell walls but also led to a relatively higher presence of double cell walls and intrahyphal hyphae (Fig. 5C a–f). Collectively, disruption or overexpression of the *FgCON7* gene resulted in abnormal production of conidia and chlamydospore-like structures, suggesting that *Fg*Con7 might function as a crucial and comprehensive regulator in the asexual development of *F. graminearum*.

### *Fg*Con7 is involved in chitin synthesis and alters cell wall integrity

**Fig 6:**
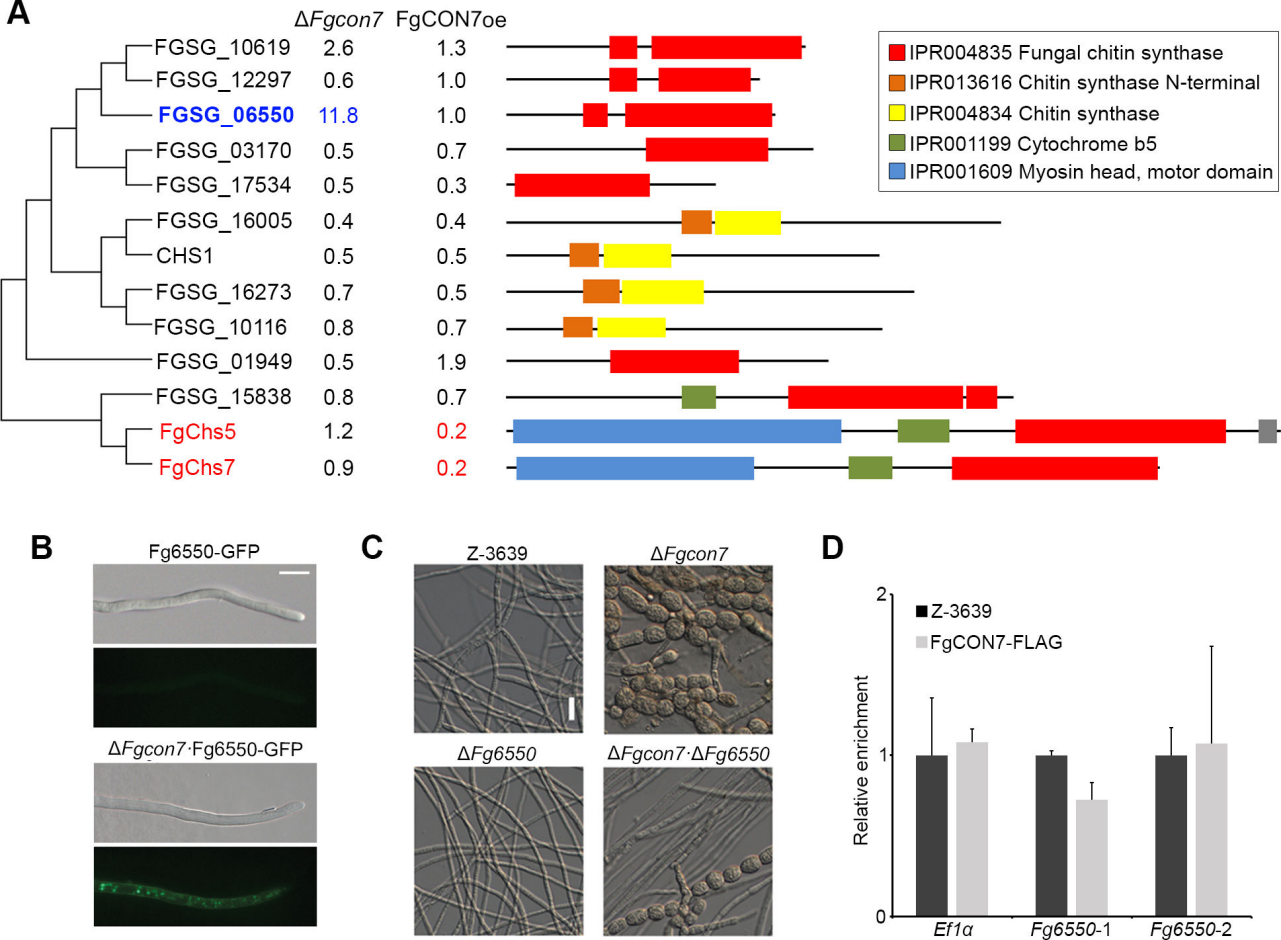
*FgCON7* affects the expression of chitin synthase. (**A**) Expression pattern of putative 13 chitin synthase genes with the phylogenetic tree and domain architecture. The alignment was performed with ClustalW, and the MEGA program Version 4.0 was used to perform a 1,000 bootstrap phylogenetic analysis using the neighbor joining method. The transcript levels of the putative chitin synthase genes in *FgCON7* deletion and overexpression mutants are indicated. The relative expression level of each gene is displayed beside the name of chitin synthase. (**B**) Microscopic image of ∆*Fgcon7*·6550-GFP strain. Microscopic observation was performed 3 days after inoculation in MM. (**C**) Scanning electron microscopy (SEM) images of mycelia of *F. graminearum* strains. (**D**) ChIP-qPCR of *Fg6550*. Two random promoter regions of selected genes were analyzed, and *EF1α* was used as the control. Data are presented as the means and standard errors from two biological replicates.

Given that chitin accumulation is a major feature of chlamydospores in Δ*Fgcon7* strains, we investigated the potential involvement of *FgCON7* in chitin synthesis, a factor crucial for asexual differentiation. To explore this concept, we performed real-time quantitative polymerase chain reaction (RT-qPCR) to examine the expression of thirteen putative chitin synthase genes in minimal media culture. These selected genes (InterPro: IPR004834, chitin synthase; IPR004835, fungal chitin synthase) were analyzed for their transcripts in the wild-type, Δ*Fgcon7*, and FgCON7oe strains (Fig. 6A). Most chitin synthase genes, including *FgCHS5* and *FgCHS7*, displayed similar or slightly decreased expression in Δ*Fgcon7* strains compared to the wild-type strain. However, a significant exception was *Fg6550* (locus ID: FGSG_06550), which showed more than a tenfold increase in expression in Δ*Fgcon7* strains. When *FgCON7* was overexpressed, crucial chitin synthases for mycelia morphogenesis (*FgCHS5* and *FgCHS7*) exhibited substantial downregulation compared to the wild-type strain (21). Since Δ*Fgcon7* strains accumulated a significant amount of chitin compared to the wild-type strain during chlamydospore-like structure formation, we proposed that *Fg*6550 may serve as a critical chitin synthase under the regulation of *Fg*Con7. To test this, we generated Fg6550-GFP and Δ*Fgcon7*·Fg6550-GFP, respectively, observing a high upregulation of Fg6550 with deletion of *FgCON7* (Fig. 6B). Moreover, a deletion mutant of *Fg6550* was generated. The double deletion mutant Δ*Fgcon7*· Δ*Fg6550* exhibited partial recovery in morphological defects caused by *FgCON7* deletion (Fig. 6C; Fig. S3). However, the overexpression of *Fg6550* in the wild-type strain did not induce any changes in mycelial morphology or the amount of chitin (Fig. S4). This implies that *Fg6550* might collaborate with other chitin synthase genes rather than functioning independently in chitin synthesis. To elucidate whether *Fg*Con7, acting as a TF, directly regulates *Fg6550*, we generated Δ*Fgcon7·*FgCON7oe-FLAG using chromatin immunoprecipitation (ChIP)-quantitative PCR (qPCR). We randomly designed primers in the promoter region of the gene. However, the results revealed that *Fg*Con7 does not directly regulate the *Fg6550* gene (Fig. 6D).

**Fig 7:**
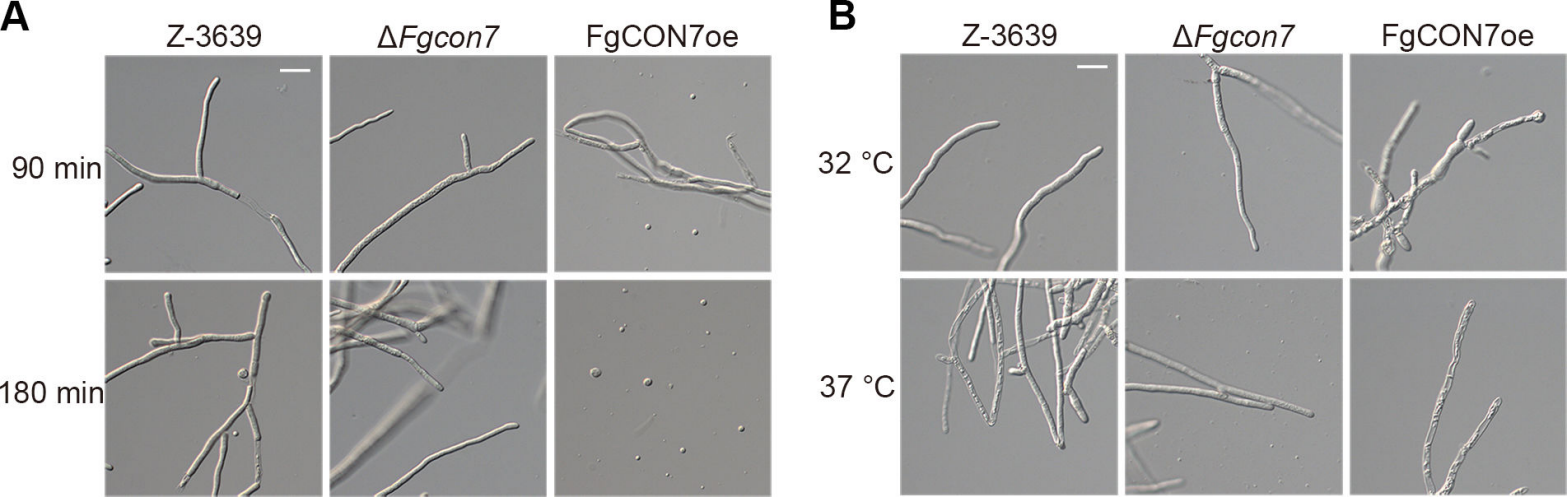
*FgCON7* contributes to cell wall integrity. (**A**) Fresh mycelia from each strain were incubated for 90 and 180 min in protoplasting solution at 30°C. (**B**) Harvested 3-day-old mycelia from YEPD media were re-inoculated in YEPD media for 24 h at 32 and 37°C. Scale bar = 20 µm.

We next investigated potential changes in the hyphal cell wall features in the Δ*Fgcon7* and FgCON7oe strains. For the cell wall integrity test, we treated 3-day-old mycelia from yeast extract peptone dextrose (YEPD) media with the cell wall-degrading enzyme mixture Driselase. In the wild-type strain, mycelial cells started collapsing after 90-min incubation, leading to the production of some protoplasts by 180 min. However, within the same incubation time, no collapsed cells or protoplasts were observed in the deletion mutant (Fig. 7A). Conversely, the FgCON7oe strain exhibited increased sensitivity to Driselase, releasing numerous protoplasts after just 90 min of incubation. Furthermore, we subjected freshly harvested mycelia in YEPD media to higher temperatures (32 and 37°C). At 32°C, approximately half of the FgCON7oe mycelia were destroyed, contrasting with the normal growth observed in the wild-type and deletion mutant strains. The *FgCON7* deletion mutant maintained normal mycelia even at 37°C, whereas about half and most of the mycelia collapsed in the wild-type and *FgCON7* overexpression strains, respectively (Fig. 7B). Collectively, these findings suggest the involvement of *FgCON7* in chitin synthesis, thereby influencing cell wall integrity.

### Conidiogenesis-related genes are directly regulated by *FgCon7*

**Fig 8:**
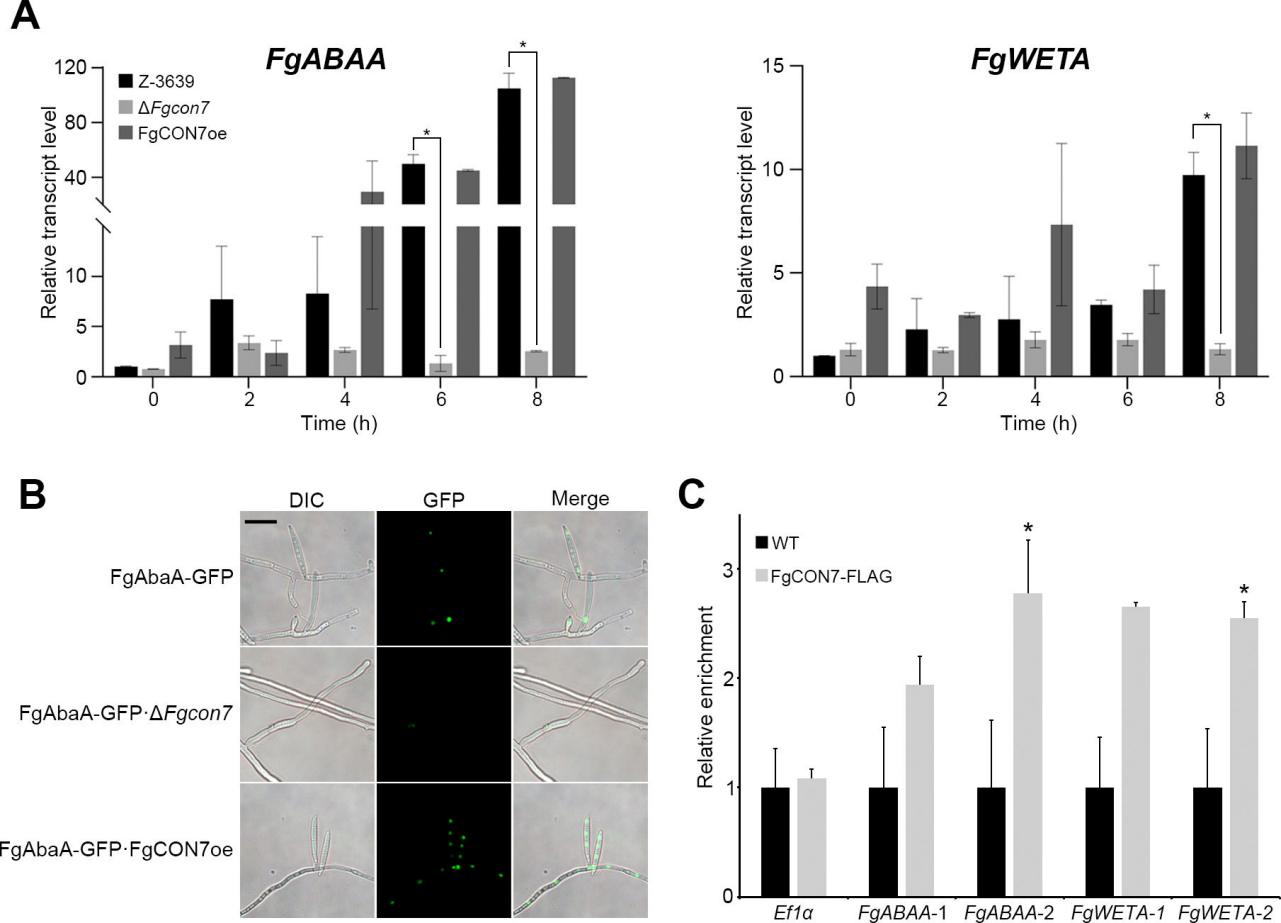
*Fg*Con7 directly regulates conidiogenesis-related genes. (**A**) Relative transcript accumulation of conidiation-related genes in *F. graminearum* strains. The transcript levels of *FgABAA* and *FgWETA* relative to *EF1α* were analyzed by quantitative real-time PCR (qRT-PCR) during the conidia induction stage in the wild-type*,* ∆*Fgcon7*, and FgCON7oe strains. Data are presented as the means and standard errors from two biological replicates. *P < 0.05. (**B**) Cellular localization of FgAbaA. FgAbaA was fused with GFP. The GFP signals were highly fluorescent in the hyphae and phalides of FgAbaA-GFP · FgCON7oe, whereas FgAbaA-GFP · ∆*Fgcon7* exhibited low fluorescence signals. (**C**) ChIP-qPCR of *FgABAA* and *FgWETA*. Two random promoter regions of selected genes were analyzed, and *EF1α* was used as the control. Data are presented as the means and standard errors from two biological replicates. *P < 0.05.

Numerous genes have been involved in conidium production, with AbaA and WetA established as master regulators orchestrating phialide formation and conidium maturation in filamentous fungi, including *F. graminearum* (22–24). Given the complete halt in conidium production upon *FgCON7* gene deletion, we aimed to explore the potential correlations between *FgCON7* and the conidiation regulators *FgABAA* and *FgWETA*. To investigate this association, we analyzed the transcript levels of *FgABAA* and *FgWETA* in the wild-type, Δ*Fgcon7*, and FgCON7oe strains during the conidium induction stage. In the wild-type strain, the *FgABAA* transcript level was upregulated at 2 h post-conidium induction, increasing until 8 h. In the Δ*Fgcon7* strain, the transcript levels of *FgABAA* and *FgWETA* were significantly decreased during conidiogenesis compared to the wild-type strain (Fig. 8A). However, in the FgCON7oe strain, the transcript level remained comparable to that of the wild-type strain during conidiogenesis. Furthermore, we found that the expression of *FgCON7* influences the fluorescence signal of FgAbaA-GFP. (Fig. 8B). These results indicate a potential genetic connection between *FgCON7* and conidiation-related genes, in both temporal and spatial aspects.

To elucidate whether *Fg*Con7, functioning as a TF, directly regulates conidiation-related genes, *FgABAA* and *FgWETA* were selected as target candidates. Meanwhile, *EF1- α* served as the nonspecific gene control. To potentially include the binding motif of *Fg*Con7, we randomly designed primers in the promoter region of the gene. As expected, the ChIP-qPCR experiments revealed higher enrichment of *FgABAA* and *FgWETA* compared to the nonspecific gene, confirming the binding ability of *Fg*Con7 to the promoter regions of these selected target genes (Fig. 8C).

**Fig 9:**
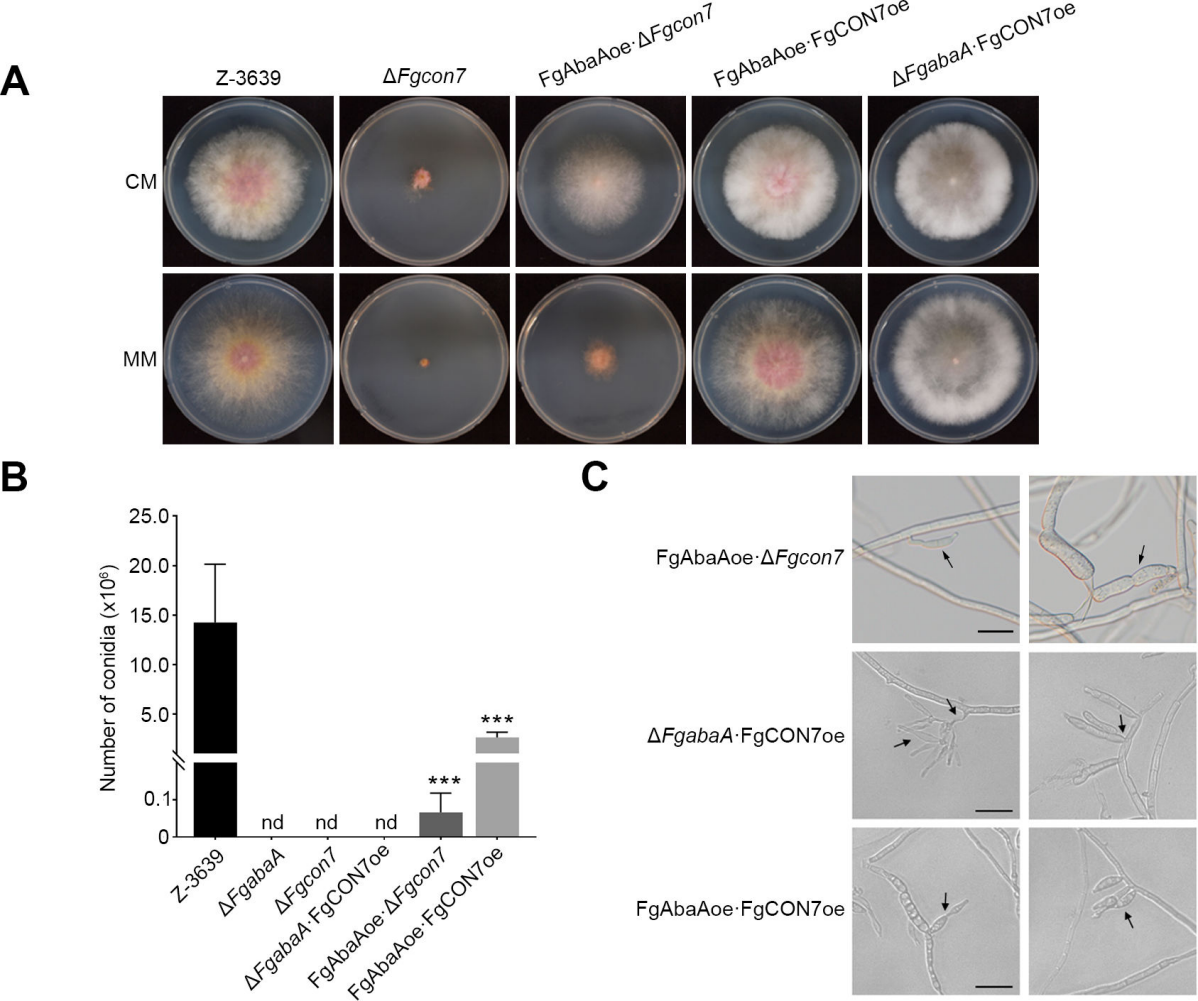
Overexpression of *FgABAA* partially rescues the ∆*Fgcon7* mutant phenotype. (**A**) Mycelial growth of *F. graminearum* strains on CM and MM. Pictures were taken 5 days after inoculation. (**B**) Quantification of conidium production and (**C**) conidial morphology in *F. graminearum* strains. Conidium production was induced in CMC media, and the number from each strain was measured 2 and 3 days after inoculation. nd: not detected.

In an attempt to identify the hierarchical relationships between *FgCON7* and the conidiation regulator *FgABAA*, we generated strains: Δ*FgabaA*·*Fg*CON7oe*,* FgABAAoe·Δ*Fgcon7*, and FgABAAoe·FgCON7oe through genetic crosses involving single-gene mutants (Fig. 9A). Interestingly, the overexpression of *FgABAA* partially alleviated the vegetative growth defect in Δ*Fgcon7* strains. Conidium production was also measured in these mutants, and it was shown that FgABAAoe·Δ*Fgcon7* strains produced a limited amount of conidia, indicating a modest recovery in conidiation (Fig. 9B). We observed that FgABAAoe·Δ*Fgcon7* strains produced thick hyphae and phialides with extremely abnormal morphology (Fig. 9C). In addition, multiple phialides in a single hypha were observed in both ∆*FgabaA*∙FgCON7oe and FgABAAoe∙FgCON7oe strains. To summarize, while sustained expression of *FgABAA* aims in partially mitigating the disruption caused by *FgCON7* deletion, overexpression of *FgCON7* does not fully restore the defect observed in Δ*FgabaA*. These findings, derived from genetic and morphological analyses, suggest that although *FgABAA* is genetically regulated by *Fg*Con7, it does not exclusively regulate the downstream pathways controlled by *Fg*AbaA.

## DISCUSSION

C2H2 zinc fingers represent one of the most prevalent DNA-binding motifs within eukaryotic genomes, commonly present in TFs (25). Studies have reported that TFs containing the C2H2 zinc finger DNA-binding motif constitute a substantial portion, ranging from 7% to 46% of the total TFs, like *Saccharomyces cerevisiae*, *Drosophila melanogaster*, *Caenorhabditis elegans*, and *Arabidopsis thaliana* (26). In fungal species, the most abundant zinc finger type is the Zn(II)_2_Cys_6_ (InterPro: IPR001138; fungal transcriptional regulatory protein), followed by the C2H2 zinc finger type (17). Within *F. graminearum*, there are potential 98 putative C2H2 zinc finger-type TFs (14%), and deletion mutants exhibited various phenotypic defects (18).

Previous studies regarding C2H2 zinc finger-type TFs in filamentous fungi have described their diverse functions, including stress responses, homeostasis, catabolite repression, and involvement in various developmental processes. This suggests that there is no direct correlation between the C2H2 zinc finger DNA-binding domain itself and their biological functions (27–31). The functions of TFs that share the same DNA-binding domain are primarily determined by the protein structure, excluding the DNA-binding region and the *cis*-regulatory elements found in downstream genes (32, 33). Therefore, characterizing an individual TF gene in a specific species holds significant value in understanding its unique properties.

Our current study demonstrated the crucial role of *Fg*Con7 as a key TF in *F. graminearum. Fg*Con7 consists of a C2H2 zinc finger DNA-binding domain and exclusive localization in nuclei, suggesting its regulatory functions. The *FgCON7* deletion mutant exhibited deficiencies across multiple physiological processes, including impaired vegetative growth, asexual sporulation, sexual development, and reduced virulence. In *Fusarium* spp., *Fo*Con7 was involved in mycelial differentiation (14), and the conidiation regulator *Fg*Htf directly regulated *FgCON7* (15). Thus, our subsequent aim was to explore the direct association between *Fg*Con7 and asexual development.

The relationship between *Fg*Con7 and chitin accumulation and the expression of chitin synthase genes is evident. In the *M. oryzae CON7* deletion mutant, the expression of class VII chitin synthase *CHS7* was reduced, leading to defects in chitin accumulation (13). Similarly, in *F. graminearum*, *FgCHS7*, a class VII chitin synthase (21), exhibited decreased expression in the *FgCON7* deletion mutant compared to the wild-type strain. This suggests that alteration of *FgCON7* expression did not induce the opposite effect on the expression of chitin synthase gene expression, such as *Fg6550* or *FgCHS5* and *FgCHS7*, implying an indirect control by *FgCON7*. We suggested that the putative chitin synthase *Fg*6550, containing a fungal chitin synthase motif (InterPro: IPR004835), might be responsible for excessive chitin accumulation in the *FgCON7* deletion mutant, considering its enhanced expression level and localization. This also influenced chlamydospore-like structure formation and impacted cell wall integrity. Although overexpression of *Fg6550* did not cause morphological changes, it is likely because *Fg6550* alone does not directly participate in chitin synthesis, similarly to the other previously reported chitin synthase genes that show no phenotypic change with a single deletion (34–36).

To our knowledge, only a few specific *F. graminearum* TFs have been identified that regulate conidium production (2, 15, 22, 37). Unlike *A. nidulans*, where the conidiation process is more intricate (22) with phialides directly forming from hyphae and continuously generating multiseptate conidia (38), *F. graminearum* exhibits a less complex conidiation process. The AbaA–WetA pathway in *F. graminearum* shares conservation similarities with *A. nidulans*, and their upstream regulators, FlbD and Htf1, have been reported (15, 22–24, 39). In this study, ChIP-qPCR analysis demonstrated that *Fg*Con7 directly regulates *FgABAA* and *FgWETA*. Additionally, considering that *Fg*Con7 is regulated by *Fg*Htf1, this study expands our understanding of the transcription pathway associated with conidiation.

A noteworthy observation is that alterations in the expression level of *FgCON7* affected cell differentiation processes, including ascosporogenesis and chlamydospore formation. The overexpression of *FgCON7* not only affected ascospore discharge but also induced changes in morphology, indicating its broader regulation of the chitin synthesis pathway in a spatiotemporal manner, thereby affecting sexual development. Furthermore, *FgCON7* deletion mutants produced a chitin-rich chlamydospore-like structure. Collectively, our data suggest that *FgCON7* serves as a multifaceted global regulator, governing vegetative growth, asexual and sexual reproduction, as well as virulence.

In conclusion, we conducted a functional characterization of the Con7 ortholog, *Fg*Con7, in *F. graminearum*, revealing extensive pleiotropic phenotypic defects upon *FgCON7* deletion. Specifically, *Fg*Con7 directly regulates conidiation-related genes like *FgABAA* and *FgWETA*, with the overexpression of *FgABAA* partially restoring the developmental defects resulting from *FgCON7* deletion. Although *Fg*Con7 is closely associated with chitin synthesis, we have yet to establish a direct genetic association with chitin synthase. Overall, our findings indicate that *FgCON7* plays distinct roles as a pivotal regulator of mycelial morphology. Further exploration into the *Fg*Con7-regulated genes, particularly regarding chitin synthesis, provides valuable insights into elucidation of the regulatory mechanisms of Con7-downstream genes.

## MATERIALS AND METHODS

### Fungal strains and media

The *F. graminearum* wild-type strain Z-3639 (40) and mutants derived from the parent strain were used in this study (Table 1). All strains were stored as conidial suspensions in 20% glycerol at –80°C. The media used in this study were prepared and used according to the *Fusarium* laboratory manual (3). The CMC medium (41), and yeast malt agar (YMA) (38) were used as previously described.

**TABLE 1:** *F. graminearum* strains used in this study

Strain	Genotype	Source or reference
Z-3639	Wild-type	(40)
HK42 (∆*Fgcon7*)	∆*Fgcon7::GEN*	This study
HK43 (FgCON7c)	∆*Fgcon7::FgCON7-GFP-HYG*	This study
mat1r	∆*mat1-1::GEN hH1::hH1-RFP-GEN*	(18)
HK47	∆*Fgcon7::FgCON7-GFP-HYG; hH1::hH1-RFP-GEN*	This study
HK68 (MoCON7c)	∆*Fgcon7::MoCON7-HYG*	This study
HK44 (FgCON7oe)	*FgCON7::GEN-P_EF1α_-FgCON7*	This study
hH1-g	*hH1::hH1-GFP-HYG*	(19)
mat1-g	∆*mat1-1::GEN hH1::hH1-GFP-HYG*	(19)
HK67	∆*Fgcon7::GEN hH1::hH1-GFP-HYG*	This study
HK45 (∆*Fg6550*)	∆*Fg6550::GEN*	This study
HK66 (Δ*Fg6550*∙Δ*Fgcon7*)	∆*Fg6550::GEN*; ∆*Fgcon7::GEN*	This study
HK46 (Fg6550-GFP)	∆*Fg6550::Fg6550-GFP-HYG*	This study
HK86 (Fg6550-GFP∙Δ*Fgcon7*)	∆*Fgcon7::GEN*; ∆*Fg6550::Fg6550-GFP-HYG*	This study
Fg6550oe	*RP27-Fg6550-GFP-HYG*	This study
Δ*Fgcon7·*FgCON7oe-FLAG	∆*Fgcon7:: GEN-P_EF1α_-FgCON7-FLAG*	This study
Δ*FgabaA*	∆*FgabaA::GEN*	(22)
FgABAAoe	*FgABAA::GEN-P_EF1α_-FgABAA*	(22)
Δ*FgabaA*·*Fg*CON7oe	∆*FgabaA::GEN*; *FgCON7::GEN-P_EF1α_-FgCON7*	This study
FgABAAoe·Δ*Fgcon7*	*FgABAA::GEN-P_EF1α_-FgABAA*; ∆*Fgcon7::GEN*	This study
FgABAAoe·FgCON7oe	*FgABAA::GEN-P_EF1α_-FgABAA*; *FgCON7::GEN-P_EF1α_-FgCON7*	This study

### Nucleic acid manipulations

Fungal genomic DNA was extracted as previously described (3). Other standard protocols for Southern blot hybridization with 32P-labeled probes were performed following standard techniques (42). Total RNA was extracted from the complete medium (CM) and minimal medium using the easy-spin Total RNA Extraction Kit (Intron Biotech, Seongnam, Korea) following the manufacturer’s instructions. The first-strand cDNA was synthesized with SuperScriptIII reverse transcriptase (Invitrogen, Carlsbad, CA). Quantitative real-time PCR (qRT-PCR) was performed with the SYBR Green Supermix (Bio-Rad, Hercules, CA, USA) and a 7500 real-time RCR system (Applied Biosystems, Foster, CA, USA). Elongation factor 1-α (Broad Institute ID: FGSG_08811.3) was used as an endogenous control for normalization, as in a previous study. The PCRs were repeated three times with two replicates per run. The PCR primers used in this study were synthesized at an oligonucleotide synthesis facility (Bioneer, Daejeon, Korea) (Table S1).

The constructs used for targeted gene deletion and complementation were generated by the double-joint (DJ) PCR method (43). For targeted gene deletion, a geneticin resistance cassette and the 5' and 3' flanking region of each target gene were amplified from pII99 and Z-3639, respectively, with appropriate primer pairs (Table S1) and fused by DJ PCR under the PCR conditions as previously described (19). To complement the Fgcon7 deletion mutant with green fluorescent protein (GFP) fusion, the deleted gene, including the promoter and the open-reading frame (ORF) was fused with GFP and the hygromycin resistance cassette (HYG) amplified with pIGPAPA-sGFP F/HYG-F1 primers from the pIGPAPA vector (44). This construct was fused with the 3' flanking region of each gene, as previously described (18). For interspecies complementation using *M. oryzae* CON7, *MoCON7* ORF which was amplified with MoCON7-F/MoCON7-R primers from previously generated cDNA of *M. oryzae* (21) was fused with the 5' flanking region of *FgCON7* and 3' flanking region-HYG amplified from genomic DNA of the FgCON7c (Δ*Fgcon7::FgCON7*) strain with FgCON7-5F/ FgCON7-5R Mo and pIGPAPA-hyg R-Mo/FgCON7-3R primers sets, respectively. The subsequent procedures for the third round of PCR and the transformation were same with complementation using the *FgCON7* gene of *F. graminearum*.

To replace the *FgCON7* promoter with elongation factor 1-α (EF1 α) promoter from *F. verticillioides* (P_EF1α_) in the wild-type strain, GEN- P_EF1α_ was amplified from the pSKGEN (45) with neo-for new and EF pro-Rev new primers, and the 5' and 3' flanking regions of the *FgCON7* gene were amplified from Z-3639 with primers FgCON7-5F/FgCON7-5R OE and FgCON7-3F OE/FgCON7-3R OE, respectively. Three fragments were fused according to the DJ PCR method (43), and the final construct was amplified with FgCON7-5N/FgCON7-3N OE primers. Resulting constructs were transformed into the wild-type or *FgCON7* deletion mutant strains, as previously described (46).

For overexpression of *Fg6550*, the *RP27-Fg6550-GFP* fusion construct was generated via the yeast gap repair approach (47). The ORF of *Fg6550* was amplified from the genomic DNA of the wild-type strain. The resulting construct and Xho1-digested pDL2 were co-transformed into the yeast strain PJ69-4A (48) using the Alkali-Cation Yeast Transformation Kit (MP bio, Santa Ana, CA, USA). The *RP27–Fg6550–GFP* fusion vector obtained from the yeast transformants was transformed into *Escherichia coli* DH10B. After verification by sequencing, the plasmid DNA was extracted with the DNA-spin Plasmid DNA Purification Kit (Intron Biotech, Seongnam, Republic of Korea) and used to transform into the wild-type strain. *Fg6550*-overexpressing strains were confirmed via qRT-PCR.

### Conidium production, fertility, and virulence testing

Conidium production was measured by counting the number of conidia produced after incubating a square (each 5 mm x 5 mm with approximately 2 mm thickness) of agar block grown on CM in 20 mL of the CMC medium.

For self-fertilization, mycelia grown on carrot agar for 5 days were removed by using the back of the surgical blade (surgical blade #11; Feather Safety Razor, Osaka, Japan) in the presence of 2.5% of sterilized Tween 60 solution (18). Female strains were spermatized with conidia suspensions (1 × 10^6^ conidia mL^−1^) of male strains for outcrosses (Table 1). After sexual induction, all of the cultures were incubated under near UV light (wavelength: 365 nm, HKiv Import and Export Co., Ltd., Xiamen, China) at 25°C.

The virulence test was performed as previously described (18). Briefly, 10 µL of the conidia suspension (1 × 10^5^ conidia mL^−1^) was injected into a center spikelet of wheat (cultivar; Eunpamil) head at mid-anthesis. After inoculation, inoculated plants were incubated in a humidity chamber for 3 days, and then head blight symptoms were checked after 11 days.

### Induction of chlamydospore-like structures

Chlamydospore-like structures were induced as previously described (20). Freshly harvested conidia and mycelia were washed twice with sterile distilled water, inoculated in 20 mL of MCMM in 250-mL Erlenmeyer flasks, and incubated at 25°C on a rotary shaker (150 rpm) under dark conditions. Conidia were prepared using YMA, and mycelia were harvested following 3 days of growth in complete medium (CM). Final conidium concentration was adjusted to approximately 1 × 10^5^ conidia mL^−1^.

### Transmission and scanning electron microscopy

Squares (each 5 mm × 5 mm with approximately 1 mm thickness) of mycelia were excised by using a surgical blade (Feather Safety Razor) from 7-day grown MM cultures, and other protocols for TEM were followed as in a previous study (49). In brief, the specimens were dehydrated in an ethanol series (30, 50, 70, and 80%) and then were embedded in London Resin White (London Resin Co., London, UK). By using a diamond knife in an ultramicrotome (MT-X; RMC, Tucson, AZ, USA), ultrathin sections were generated. Samples were stained with 2% uranyl acetate and Reynolds’ lead citrate, each for 7 min (50). Sections were visualized using an energy-filtering transmission electron microscope LIBRA 120 (Carl Zeiss, Oberkochen, Germany), operating at an accelerating voltage of 120 kV. Zero-loss energy-filtered images were recorded with a 4 K slow-scan charge-coupled device camera (4000 SP; Gatan, Pleasanton, CA, USA).

For scanning electron microscopy (SEM) observation, same samples with TEM were fixed, post-fixed, and dehydrated same as in the TEM procedure. After drying with liquid carbon dioxide, samples were mounted on a metal stub and sputter-coated with gold. The specimens were examined with a Schottky-type field emission scanning electron microscope (Supra 55VP; Carl Zeiss, Oberkochen, Germany) operated at an accelerating voltage of 2 kV.

### Histological visualization by chitin staining

Freshly harvested mycelia from 2-day-grown MM cultures were observed using a DE/Axio Imager A1 microscope (Carl Zeiss) with bright-field optics or appropriate filter sets for fluorescent visualization, as previously described (20). Chitin staining was performed by adding 2 µL of calcofluor white stock solution (10 mg mL^–1^; Sigma, 18909) to each 20 µL of the mycelial sample on slide glasses. Following 15-min incubation at 4°C, mycelia were observed. The strains containing hH1-GFP were visualized with filter set 38HE (excitation 470/40; emission 525/50) (19). The filter set 49 (excitation 356; emission 445/50) was used for visualization of calcofluor white.

### ChIP-qPCR

For ChIP experiments, the fungal mycelium was incubated in 50 mL of cross-linking buffer (0.4 M sucrose, 10 mM Tris-HCl, pH 8.0, 1 mM PMSF, and 1% formaldehyde) for 15 min, and the cross-linking was stopped by adding 2.6 mL 2 M glycine under shaking for 5 min. Mycelium pellets were collected by vacuum filtration and ground with liquid nitrogen. The powder was resuspended in 4 mL nuclei lysis buffer (250 mM HEPES, pH 7.5, 150 mM NaCl, 1 mM EDTA, 1% Triton X-100, 0.1% sodium deoxycholate, 10 mM DTT, adding a protease inhibitor cocktail) and incubated at 4 ℃ for 1 h. The samples were divided into eight aliquots of 500 µL and sonicated for 32 min. After centrifuging at 12,000 rpm for 10 min, the supernatant was collected, and immunoprecipitation was conducted using anti-FLAG magnetic beads (Sigma, M8823). Crosslinks were reversed by adding 5 M NaCl, and DNA was precipitated after treatment with RNase A and proteinase K, as previously described (51). The DNA sample was diluted 10-fold and used for qPCR. Primers were randomly designed to be located in the promoter region of the putative binding genes, *FgABAA*, *FgWETA*, and *Fg6550*. The enrichment level was determined using the 2^−ΔΔCT^ method (52), and the EF1 α was used as an internal control. The experiment was performed with two biological replicates, and primers are listed in Table S1.

### Cell wall integrity test

Cell wall integrity tests of *F. graminearum* strains were followed as per a previous study (53). Because the *FgCON7* deletion mutant did not produce conidia, we used fresh mycelia as inocula in these experiments. Harvested 3-day-old mycelia from YEPD (0.3% yeast extract, 1% peptone, and 2% dextrose) media were re-inoculated in YEPD media for 24 h at 32 and 37°C on a rotary shaker (150 rpm) for temperature sensitivity testing. For the cell wall degradation test, 3-day-old mycelia were incubated with Driselase (20 mg/mL) (Karlan Research Products, Santa Rosa, CA, USA) in 1.4 M KCl at 30°C.

## Data Availability

The data that support the findings of this study are available on request from the corresponding author.

## SUPPLEMENTAL MATERIAL

Supporting information msphere.00818-23-s0001.docx Table S1 and Fig. S1-S4.

ASM does not own the copyrights to Supplemental Material that may be linked to, or accessed through, an article. The authors have granted ASM a non-exclusive, world-wide license to publish the Supplemental Material files. Please contact the corresponding author directly for reuse.
