# Supplementary material for: Con7 is a key transcription regulator for conidiogenesis in the plant pathogenic fungus Fusarium graminearum
Source: mSphere. 2024 Apr 9;9(5):e00818-23. doi: 10.1128/msphere.00818-23 (PMC11237738; doi:10.1128/msphere.00818-23)
Supplement: Supporting information — Table S1 and Fig. S1-S4. [file msphere.00818-23-s0001.docx]

Supporting information

Table S1. Primers used in this study

| **Primer** | **Sequence (5’ → 3’)** | **Description** |
| --- | --- | --- |
| FgCON7-5F | CAGCACGCAGCAAGTAAATCCAT | Forward and reverse primers for amplification of 5’ flanking region of *FgCON7* with tail for the geneticin resistance gene cassette fusion |
| FgCON7-5R | gcacaggtacacttgtttagagGCTCCACGGGGCTGATGATAA |  |
| FgCON7-3F | ccttcaatatcatcttctgtcgGATGGTCAAGCAAGGATCAAGGTTC | Forward and reverse primers for amplification of 3’ flanking region of *FgCON7* with tail for geneticin resistance gene cassette fusion |
| FgCON7-3R | GGTGTAAGCAAGTGAGCCGAGTGT |  |
| FgCON7-5N | TCCTTTTTGCTTGGGCTCCTTCAT | Forward and reverse nest primers for third fusion PCR for amplification of *FgCON7* deletion construct |
| FgCON7-3N | CCCTATCCTTCGGTATCCCAGTTGC |  |
| 6550-5F | GTGGCTTGCATATTTACGTGTTTGAA | Forward and reverse primers for amplification of 5’ flanking region of *Fg6550* with tail for the geneticin resistance gene cassette fusion |
| 6550-5R | ccttcaatatcatcttctgtcgTGCTGGACAGACATGTTTCCTCTCT |  |
| 6550-3F | gcacaggtacacttgtttagagGTGGAACACACTAAACAACGATGCA | Forward and reverse primers for amplification of 3’ flanking region of *Fg6550* with tail for geneticin resistance gene cassette fusion |
| 6550-3R | GGAGAGTTGACCACCGCCATAA |  |
| 6550-5N | GCTGTCGTGCTATGCGGAGTC | Forward and reverse nest primers for third fusion PCR for amplification *Fg6550* deletion construct |
| 6550-3N | AGCGATTGGAAGAGCAGAGTGAAC |  |
| Gen-For | CGACAGAAGATGATATTGAAGG | Forward and reverse primers for amplification of geneticin cassette from pII99 vector |
| Gen-Rev | CTCTAAACAAGTGTACCTGTGC |  |
| FgCON7-5R GFP | gaacagctcctcgcccttgctcacGTGGCTTGGGGGTTGCG | Reverse primer for amplification of 5’ flanking region of *FgCON7* with a tail for GFP tagging complementation |
| FgCON7-3F GFP | tttcataccacacctgcccaccGATGGTCAAGCAAGGATCAAGGTTC | Forward primer for amplification of the 3’ flanking region of *FgCON7* with a tail for GFP tagging complementation |
| 6550-5R GFP |  | Reverse primer for amplification of 5’ flanking region of *Fg6550* with a tail for GFP tagging complementation |
| 6550-3F GFP |  | Forward primer for amplification of the 3’ flanking region of 6550 with a tail for GFP tagging complementation |
| pIGPAPA-sGFP F | GTGAGCAAGGGCGAGGAGCTG | Forward and reverse primers for amplification of the *GFP-HYG* construct from pIGPAPA vector |
| HYG-F1 | GGCTTGGCTGGAGCTAGTGGAGG |  |
| FgCON7-5R OE | gatagtggaaaccgacgccccTGCTGAGAGCGACTCGCG | Reverse primer for amplification of 5’ flanking region of *FgCON7* with tail for overexpression |
| FgCON7-3F OE | cacaaaaggaacccaatcttcaaagATGTCTTTGGTGCCAACACAGC | Forward and reverse primer for amplification of 3’ flanking region of *FgCON7* with tail for overexpression |
| FgCON7-3R OE | GTGGCTTGGGGGTTGCG |  |
| FgCON7-5R-Mo | gcgtgacgaggccaacatactaGCTCCACGGGGCTGATGATAA | Reverse primer for amplification of 5’ flanking region of *FgCON7* with tail for *Magnaporthe oryzae* *CON7* complementation |
| MoCON7-F | TAGTATGTTGGCCTCGTCACGC | Forward and reverse primers for amplification of *M. oryzae* *CON7* cDNA |
| MoCON7-R | TCAGATCGAGCCACGGTTAGTG |  |
| Neo-For new | GGGGCGTCGGTTTCCACTATC | Forward and reverse primers for amplification of the *GEN-P_EF1α_* from the pSKGEN vector |
| EF Pro-Rev new | CTTTGAAGATTGGGTTCCTTTTGTGATA |  |
| Neo-For 5N | GGCGAGTACTGCCAGCAGTAGACAC | Forward and reverse nest primers for third fusion PCR for amplification of the *GEN-P_EF1α_-FgCON7* construct |
| FgCON7-3N OE | AGCAGGTTGGTAGCCGATGG |  |
| UBH-RT-F | GTTCTCGAGGCCAGCAAAAAGTCA | For real-time PCR of *UBH1* |
| UBH-RT-R | CGAATCGCCGTTAGGGGTGTCTG |  |
| EF1α-RF-F | GGCTTTCACCGACTACCCTCCTCT | For real-time PCR of *EF1α* |
| EF1α-RF-R | ACTTCTCGACGGCCTTGATGACAC |  |
| FgCON7-RT F | GGTGGTGCGCCGTTGACTC | Forward and reverse primers for qRT-PCR of the *FgCON7* gene |
| FgCON7-RT R | GCCTTTTCGCAGCCGTTCC |  |
| AbaA-RT-F | ACGCAAGCAAGTCTCAAGTCATA | For real-time PCR of *ABAA* |
| AbaA-RT-R | TGTTCCTCCTCGTCATAGTAATCA |  |
| WetA-RT-F | GTTCCAGGTACTCCCACTGCCAT | For real-time PCR of *WETA* |
| WetA-RT-R | ACGTTCTCGTCGCGCTTTGGT |  |
| ChIP-EF-F | CGTCAGTGCAGCTTCAGGTG | For ChIP-qPCR of *EF1 α* |
| ChIP-EF-R | TCAACAGAGGGAGGACACGAT |  |
| ChIP-ABAA-F2 | CCCAACACACGCCCCTCC | For ChIP-qPCR of *ABAA* |
| ChIP-ABAA-R2 | GGGGCCGCGACGACTATC |  |
| ChIP-ABAA-F3 | GACTGGTAAGGGAAGGAGTAGGGT | For ChIP-qPCR of *ABAA* |
| ChIP-ABAA-R3 | CTCCTCCATACTCAACCGTCCATC |  |
| ChIP-WETA-F1 | GAGCCAGAATGGATTCGCAGAC | For ChIP-qPCR of *WETA* |
| ChIP-WETA-R1 | TTTAACGGACAAGACGGCAACAT |  |
| ChIP-WETA-F2 | CCTTTCACCTCCCCTCCTCAA | For ChIP-qPCR of *WETA* |
| ChIP-WETA-R2 | TGTATGGAGCGTTTGATTTGATGG |  |
| ChIP-6550-F | GAAGCAAGTTTGGAACTGAGAACG | For ChIP-qPCR of *Fg6550* |
| ChIP-6550-R | ACGAGGGGCGTACCGGTCT |  |
| ChIP-6550-F2 | GTGCAAAGAATTAGGAACAAACGG | For ChIP-qPCR of *Fg6550* |
| ChIP-6550-R2 | AAACAACAAAGCGCAATAATCAGG |  |
| 15838-RT F | TATGGCCATTGTCGGGTTTCTCAC | Forward and reverse primers for qRT-PCR of the *15838* gene |
| 15838-RT R | GGGCCCAACCCGTCACTCC |  |
| 01949-RT F | TCACCTCGACGACAAAGAAACACA | Forward and reverse primers for qRT-PCR of the *01949* gene |
| 01949-RT R | GAGAAGCGCAGCATGGTCAAAG |  |
| 01964-RT F | CCGAGAACCAGGCCATTGTCTTT | Forward and reverse primers for qRT-PCR of the *CHS5* gene |
| 01964-RT R | GTGTTGTGGCGGTCTTTGTAGTTG |  |
| 16005-RT F | CGTAACGAAAGATGGGTCCTCAAG | Forward and reverse primers for qRT-PCR of the *16005* gene |
| 16005-RT R | GGGCGAAAGTATGATCTGTGAACC |  |
| 03170-RT F | TGCGCGGGACAAGGATGCT | Forward and reverse primers for qRT-PCR of the *03170* gene |
| 03170-RT R | GTGATACAACCAGGCAGACAAGTGAC |  |
| 16273-RT F | GGTGTCTATCAGGATGGCGTTCTC | Forward and reverse primers for qRT-PCR of the *16273* gene |
| 16273-RT R | ATAAACTGCACGGGAGGGAGATTG |  |
| 06550-RT F | CTGCTGAGCCGGAGAAGATGG | Forward and reverse primers for qRT-PCR of the *06550* gene |
| 06550-RT R | TCGTGATGGGCCTGGTTCG |  |
| 10116-RT F | AACCAGCAGCTCATCCGACCTACA | Forward and reverse primers for qRT-PCR of the *10116* gene |
| 10116-RT R | TGGTACCCGCATCAAGCAGAAT |  |
| 10327-RT F | CGCTAAGAAGATCAACTCGCACAG | Forward and reverse primers for qRT-PCR of the *10327* gene |
| 10327-RT R | ACCACAGGCACCACCGCACAT |  |
| 10619-RT F | CAAAACCAGGGCAAGCGTGACA | Forward and reverse primers for qRT-PCR of the *10619* gene |
| 10619-RT R | TCGCTAATGCACTGGTCCTCAAAG |  |
| 17534-RT F | CCGTCAGGTCATGGTCGTTATTCT | Forward and reverse primers for qRT-PCR of the *17534* gene |
| 17534-RT R | GTCCTTTTTGCCGCTGTTTCTGA |  |
| 15914-RT F | GACACTTTGGGCATTAACGCTGAG | Forward and reverse primers for qRT-PCR of the *CHS7* gene |
| 15914-RT R | GCAGCCTTCTCTAAAATGACTTCTCG |  |
| 12297-RT F | GCCTCCATCATCTCCATTTCCATC | Forward and reverse primers for qRT-PCR of the *12297* gene |
| 12297-RT R | CCACGGACACGACCATCACAGA |  |


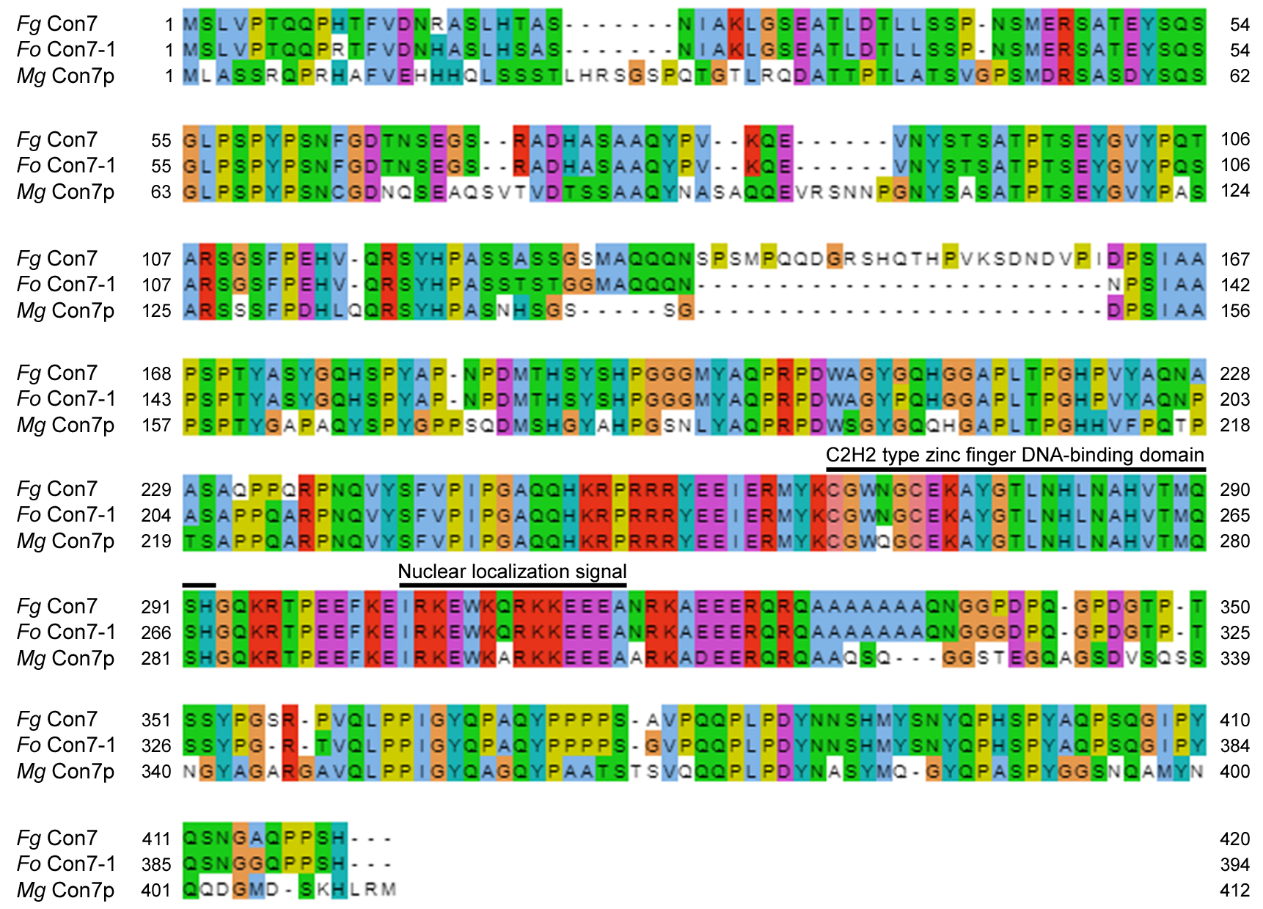


**Figure S1**. Alignment of *Fg*Con7 and *Mo*Con7. Amino acid alignments were made by using the CLUSTALX program.


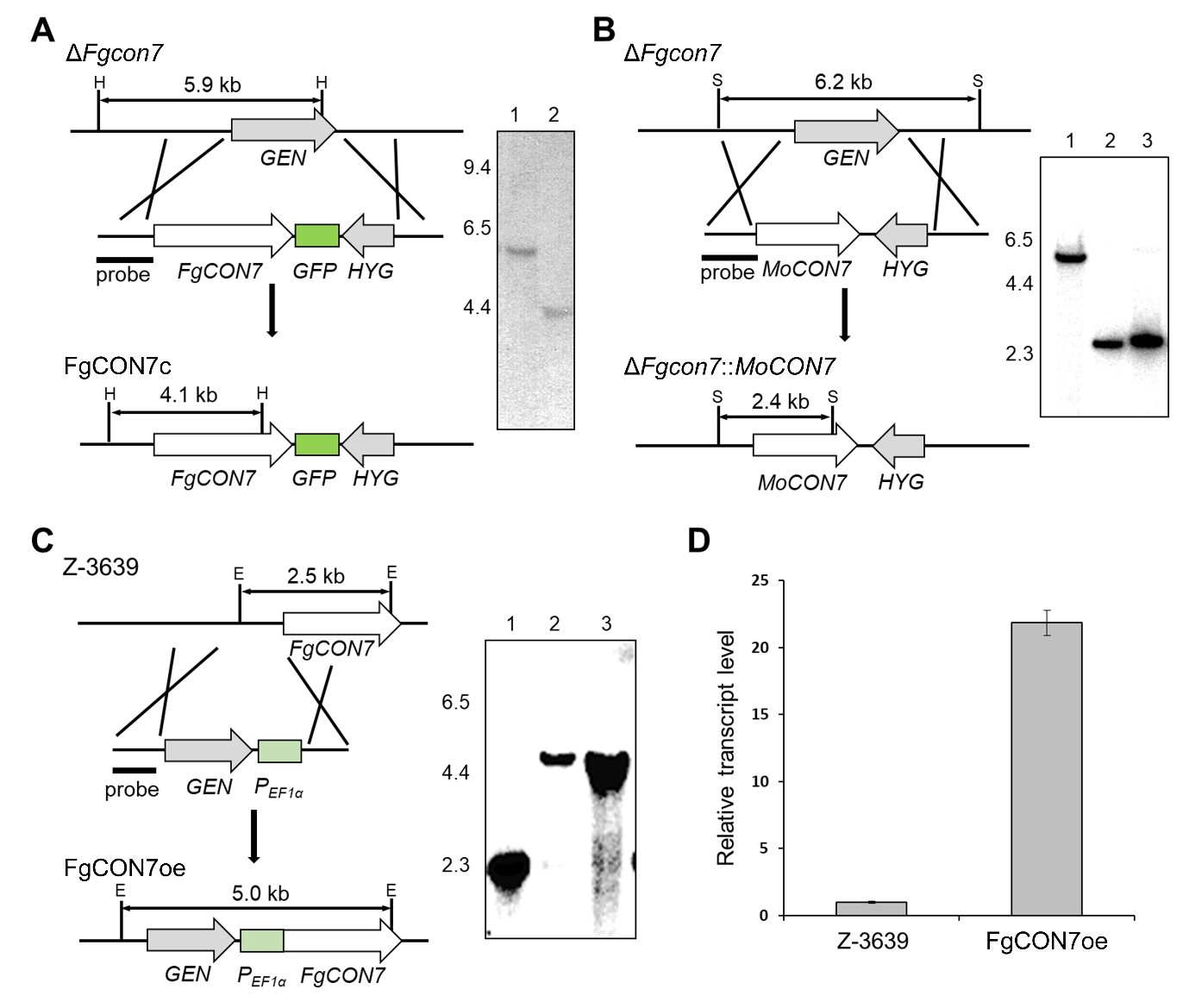


**Figure S2**. Complementation and overexpression of *CON7* in *F. graminearum*. (A) Complementation of *CON7*. Lane 1, *con7* deletion mutant HK42; lane 2, HK42*-*derived strain complemented with *FgCON7-GFP*. (B) Interspecies complementation of *M. oryzae* *CON7* in the *Fgcon7* deletion mutant. Lane 1, deletion mutant; lane 2 and 3, complemented strain HK68. (C) The *FgCON7* promoter was replaced with the *EF1α* promoter. The left and right panels show the strategy of HK44 strain construction and Southern hybridization, respectively. Lane 1, wild-type strain Z-3639; lane 2 and 3, complemented strain HK68. (D) Relative transcript accumulation of *FgCON7* in wild-type and *FgCON7*-overexpressed strains. Total RNA of each strain was extracted 3 days after inoculation in complete medium (CM). The transcript levels of *FgCON7* were analyzed by quantitative real time-PCR (qRT-PCR). H, HindIII; S, SaclI; *GEN*, geneticin resistance gene cassette; *HYG,* hygromycin B resistance gene cassette. The sizes of DNA standards (kb) are indicated on the left of each blot.


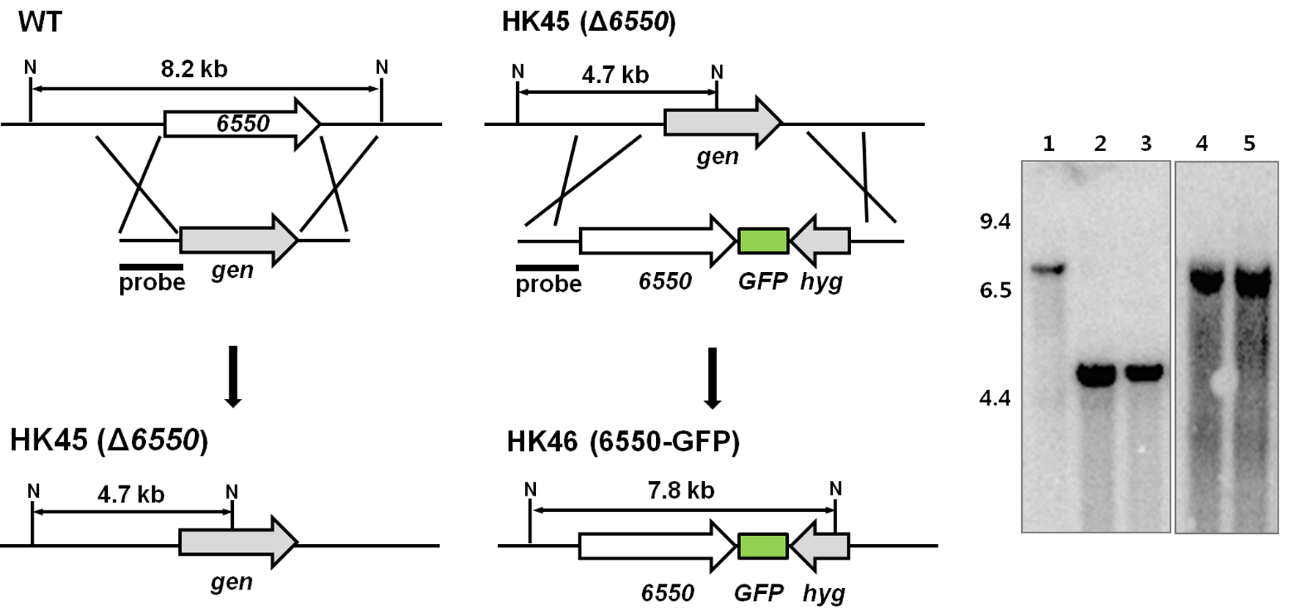


**Figure S3**. Deletion of *Fg6550* in *F. graminearum*. Lane 1, Z-3639; land 2 and 3, HK45; lane 4 and 5, HK 46. *con7* deletion mutant HK42; lane 2, HK42*-*derived strain complemented with *FgCON7-GFP*. The sizes of DNA standards (kb) are indicated on the left of each blot.


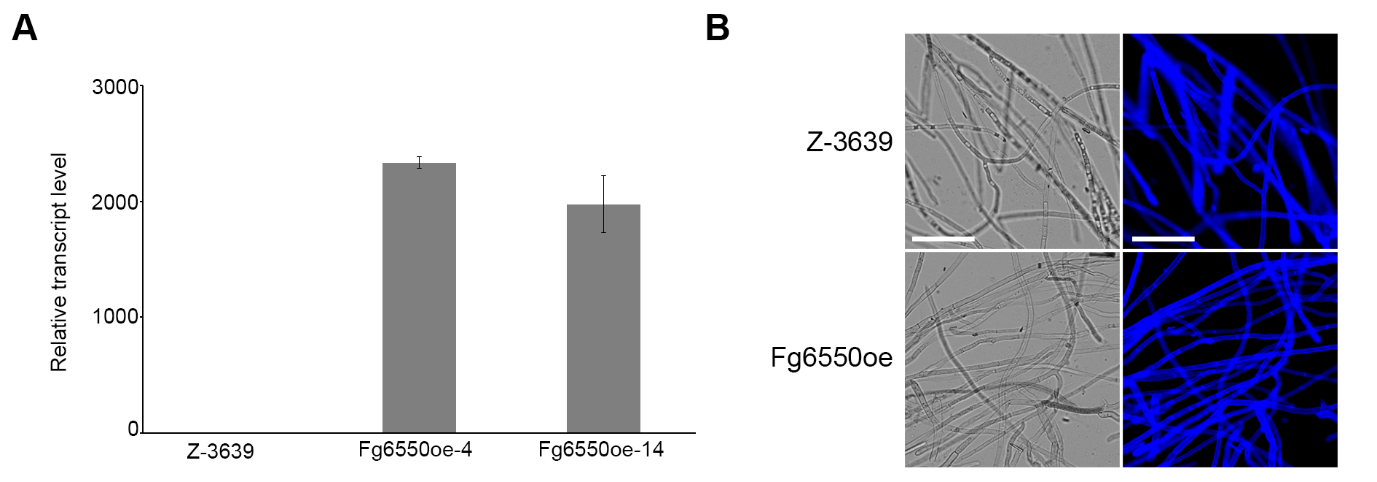


**Figure S4.** Overexpression of *Fg6550* in *F graminearum*. (A) Relative transcript accumulation of *FgCON7* in wild-type and *Fg6550*-overexpressed strains. Total RNA of each strain was extracted 2 days after inoculation in complete medium (CM). The transcript levels of *Fg6550* were analyzed by quantitative real time-PCR (qRT-PCR). (B) Histological visualization of mycelia. Chitins were stained with Calcofluor white. Scale bar = 50 µm.
